# Spontaneous Regression of an Incidental Spinal Meningioma

**DOI:** 10.3889/oamjms.2016.005

**Published:** 2015-12-21

**Authors:** Ali Yilmaz, Zahir Kizilay, Ahmet Sair, Mucahit Avcil, Ayca Ozkul

**Affiliations:** 1 *Adnan Menderes University, Faculty of Medicine, Neurosurgery Department, Aydin, Turkey*; 2 *Adnan Menderes University, Faculty of Medicine, Neurology Department, Aydin, Turkey*; 3 *Adnan Menderes University, Faculty of Medicine, Department of Emergency Medicine, Aydin, Turkey*

**Keywords:** meningioma, spinal cord, tumour regression

## Abstract

**AIM:**

The regression of meningioma has been reported in literature before. In spite of the fact that the regression may be involved by hemorrhage, calcification or some drugs withdrawal, it is rarely observed spontaneously.

**CASE REPORT:**

We report a 17 year old man with a cervical meningioma which was incidentally detected. In his cervical MRI an extradural, cranio-caudal contrast enchanced lesion at C2-C3 levels of the cervical spinal cord was detected. Despite the slight compression towards the spinal cord, he had no symptoms and refused any kind of surgical approach. The meningioma was followed by control MRI and it spontaneously regressed within six months. There were no signs of hemorrhage or calcification.

**CONCLUSION:**

Although it is a rare condition, the clinicians should consider that meningiomas especially incidentally diagnosed may be regressed spontaneously.

## Introduction

Meningiomas are the most common non-glial tumors of the brain and spine and they account for approximately 13-20% of all primary intracranial neoplasms [1]. They are benign tumors usually arising from the meningothelial cells or arachnoid cap cells which reside in the arachnoid layer covering the surface of the brain [2]. They may occur intracranially or within the spinal canal and less than 10% ever cause clinical symptoms. The majority of meningiomas are sporadic, although several associations exist with 10% of multiple meningiomas associated with neuro fibromatosis type 2 [3].

In literature it has been previously reported that regression of meningioma can be seen due to hemorrhage, calcification and drug withdrawal [4-7]. However, spontaneous regression of meningioma is rarely observed [8].

Here we report a case of incidentally diagnosed spinal meningioma which regressed within six months without any hemorrhage or drug withdrawal.

## Case Report

**Figure 1:**
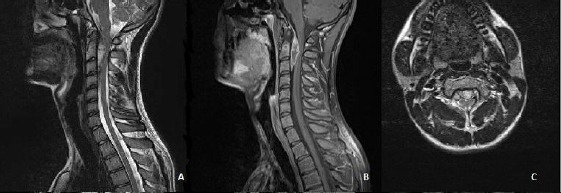
*The cervical MRI findings sagittal T2 weighted (A), T1 weighted sagittal (B) and axial (C) images with contrast enhancement showing extradural, cranio-caudal lesion concominant with meningioma at C2-C3 levels of the cervical spinal cord*.

A 17-year-old male patient with a history of dizziness and an incidentally diagnosed meningioma was admitted to our neurosurgery clinic. His medical history was unremarkable and he wasn’t taking any kind of treatment. His neurological examination was completely normal. The routine laboratory investigation revealed no pathology. The cervical MRI showed an extradural, cranio-caudal contrast enchanced lesion at C2-C3 levels of the cervical spinal cord. The lesion had a length of 38.8 mm with 11.3 mm a sagittal diameter. It appeared as an isointense mass on the T1-weighted sequence, slightly hyperintense on the T2 sequence. The lesion showed contrast enhancement on a postgadolinium T1-weighted sequence as well. There is a slight compression towards the cervical spinal cord. These imaging characteristics strongly favored the diagnosis of a spinal meningioma (Figure 1A,B,C).

**Figure 2:**
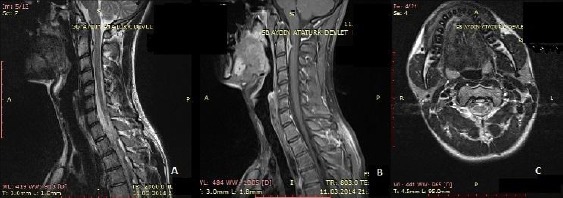
*The control cervical MRI showing regression of meningioma in T2 weighted (A), T1 weighted sagittal (B) and axial (C) images*.

There were no neurological deficits or subjective complaints related to the tumor. Since he had no complaints and didn’t want any kind of surgical approach, the meningioma was followed by a control MRI. After three months the control cervical MRI revealed the regression of the meningioma (Figure 2 A,B,C).

**Figure 3:**
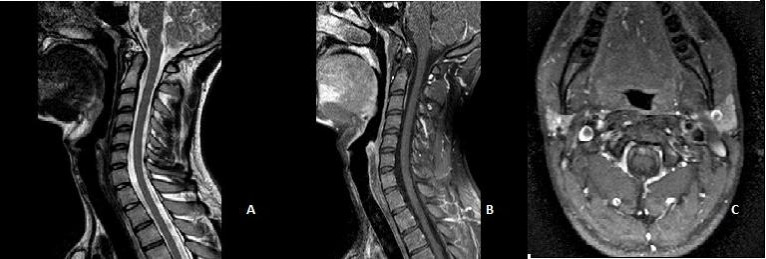
*After six months cervical MRI showed complete regression of meningioma in T2 weighted (A), T1 weighted sagittal (B) and axial (C) images with lineer contrast enchancement without any solid lesion*.

The length of the lesion regressed to 35 mm with a sagittal diameter of 6 mm without any signs of hemorrhage and calcification. In his third MRI after six months the lesion was completely regressed (Figure 3A,B,C).

There was only a linear contrast enhancement without any signs of a solid lesion. During this period he had got no kind of medical treatment.

## Discussion

The typical meningioma is a dural-based, markedly enhancing extra-axial mass. It may orginate from meningothelial cells whether intracranial, spinal or ectopic. Their common intracranial localisations are parasagittal, and sphenoid regions (75%). They can be also seen in spine (12%), cerebellopontine angle (2-4%), intraventricular (2-5%), orbital (1%) and ectopic (<1%) [1]. Although the definite treatment for symptomatic meningiomas is complete surgical resection, less common locations and asymptomatic meningiomas are important for the diagnosis and the appropriate treatment. Our patient had a spinal meningioma which is less common than intracranial ones. It was asymptomatic and incidentally diagnosed. We presented our case for being a rare asymptomatic spinal meningioma showing regression within six months without anykind of treatment.

Regression can be seen in both malign and benign tumors. The underlying mechanisms are associated with mainly related to the apoptosis and activity of the immune system, and the microenvironment of the tumor. The oncogenic suppressors of DNA are also related to this process [9]. Reports of spontaneous regression of meningiomas in the literature are rare. In literature spontaneous regression of meningiomas are reported in association with intratumoral hemorrhage [4] and progestative hormonal treatment withdrawal [5-7]. It can also gradually shrink together with a decrease of edema and increase of calcification [8]. The intratumoral hemorrhage and calcification may cause necrosis of the tumor.

In literature it has been also reported that the administration of medroxyprogesterone acetate can cause the growth of multiple meningiomas. As expected, hormonal withdrawal is associated with spontaneous regression [10]. This has been also reported in association with cyproterone acetate withdrawal [5]. It is also wellknown that meningiomas may get smaller in size after pregnancy. Shimizu et al also reported a similar regression of meningioma during a 2-year period due to chlormadinone acetate (a progesterone agonist) which was used for the treatment of benign prostatic hyperplasia [6].

Meningiomas are known to cause local vascular disturbances, mainly venous congestion and enhanced circulation. The decrease in dural blood supply and venous drainage may also contribute the regression process. The hemodynamic alterations may have contributed to vessel and tissue ischemia and, consequently, led to tumor hemorrhage and eventual necrosis [11].

All these data reinforces the link between meningioma regression and hormonal status, hemorrhage and calcification. However our patient had an incidentally diagnosed meningioma without any symptoms. He refused any kind of treatment and he hadn’t been using any medication previously. He had no hemorrhage and vascular pathology either. It is difficult to explain the underlying mechanism of the resolution of meningioma since his medical history was unremarkable.

The resolution of meningiomas due to intratumoral hemorrhage may be seen within 7 years [4] or in a shorter period of time (6 months - 2 years) in some cases of drug withdrawal [6, 10]. In our patient the meningioma at C2-C3 levels of the cervical spinal cord disappeared within 6 months. The regression of the tumor could be seen in repeated MRIs. To our knowledge this is the first spinal meningioma which was regressed spontaneously without any clinical symptoms. Additionally we couldn’t find any reasons to explain the regression. There had been no signs of hemorrhage or calcification. Our patient was male and had no previously treated with drugs that may cause growth or regression of a meningioma. To date, there have been no reports showing spinal meningioma regression in such a short time without any symptoms.

In conclusion, according to the present data the regression of meningioma may not be uncommon in cases of intratumoral hemorrhage, calcification or drug withdrawal such as medroxyprogesterone acetate, cyproterone acetate and chlormadinone acetate. Our case had none of these factors related to regression of meningioma. We believe that the factors related to tumoral growth and regression is still unclear and further studies would be beneficial to clarify the mechanisms and factors related to spontaneous regression.
